# Individualized blended care for patients with colorectal cancer: the patient’s view on informational support

**DOI:** 10.1007/s00520-020-05810-5

**Published:** 2020-10-12

**Authors:** Anna Sigridur Islind, Victoria Johansson, Helena Vallo Hult, Pia Alsén, Emma Andreasson, Eva Angenete, Martin Gellerstedt

**Affiliations:** 1 Department of Computer Science, Reykjavik University, Reykjavik, Iceland; 2 School of Business, Economics and IT, University West, Trollhättan, Sweden; 3 Region Västra Götaland, NU Hospital Group, Trollhättan, Sweden; 4 Department of Health Sciences, University West, Trollhättan, Sweden; 5 Department of Surgery, SSORG - Scandinavian Surgical Outcomes Research Group, Institute of Clinical Sciences, Sahlgrenska Academy, University of Gothenburg, Gothenburg, Sweden; 6 Region Västra Götaland, Sahlgrenska University Hospital/Östra, Department of Surgery, Gothenburg, Sweden

**Keywords:** Blended Care, Cancer, Colon cancer, Communication, Information, Patient information, Patient satisfaction

## Abstract

**Purpose:**

The number of colorectal cancer patient survivors is increasing. Information and support during and after treatment are requested by patients, but questions remain on what to provide. The aim of this study was to understand what informational needs colorectal cancer patients and survivors have, with a focus on the potential support given by patient peers and the use of blended care.

**Methods:**

A qualitative study using focus groups was conducted with patients diagnosed at the same hospital at least one year prior to the initiation of the study. The focus group interviews were transcribed verbatim and analyzed using deductive content analysis.

**Results:**

The need for informational support varied over time and depended on individual patient characteristics. Timing was crucial and patients requested options of blended care and informational support after treatment cessation. The patients felt alone after treatment and requested assistance in communication with their next-of-kin. They also identified the value of peer support, especially to contextualize knowledge provided by healthcare.

**Conclusion:**

This study showed a need for focus on individualized informational support. Blended care through integrating communication with peers online could be one way to support patients, both to enable shared decision-making as well as to provide person-centered care.

## Introduction

Psychosocial interventions, like cognitive behavioral therapy, information/education, and social group therapy, have been shown beneficial to improve quality of life (QoL) among colorectal cancer (CRS) survivors, but are underused in clinical practice [1, 2]. Furthermore, eHealth is suggested as a potential way of improving support requested by patients and enhancing person-centered care [3]. There is a need to better understand the use of such interventions, especially among patients with colorectal cancer. Physical changes and the diagnosis in itself have an impact of QoL in terms of emotions and behavioral functioning, but there are available interventions, and if well aligned and adjusted to patient needs, these could help patients adapt [4].

It is noteworthy that despite the evidence, patient use of psychosocial services may be as low as 8%, even given a referral rate of 70% [5]. One of the reasons reported is that patients say “No need/have all support I need elsewhere” [5, 6]. Other barriers reported are lack of information, logistics (e.g., inconvenient time and location or transportation), and low confidence in effect [5]. Remarkably, and in contradiction to the perception of “no need,” many patients report psychosocial and psychological needs as unmet needs [7]. There is certainly a call for clarifying patient perception regarding the need for psychosocial care among patients with cancer [6]. The research focusing on support groups is scarce [8].

Many patients seem to request more information, education, and improved communication [9]. A change of traditional care into blended care, defined as the integration of eHealth and traditional care [10], for instance self-management programs supported by eHealth, may offer a way to reach person-centered care [11]. One way of delivering blended care would be to use health platforms with educational content, but also digital meeting places intended for peer support with or without professional guidance. There is, however, limited knowledge about the exact nature of these needs and how these could be supported, especially for patients with colorectal cancer. To improve healthcare, more knowledge and understanding is required.

The primary aim of this study is to achieve a deeper understanding of the support requested and given for patients with colorectal cancer. A secondary aim of this study is to discuss the suitability of blended care for patient support.

## Method

The data gathering for this paper was based on qualitative focus group interviews [12]. The focus groups included patients with different backgrounds, ranging in age, and all had finished their primary treatment for colorectal cancer. The participants were treated at Sahlgrenska University Hospital in Sweden at least 1 year prior to the study. They were identified by nurses at the outpatient clinic and contacted by with an introductory letter followed by a telephone call. They received written information and signed informed consent (see EPN 262-18). All patients were a part of follow-up care; one patient was in palliative care. They were divided into three focus groups and each focus group session lasted 2 h. The study aimed for heterogeneity in diagnosis and gender when dividing the patients into focus groups. However, in focus group three, the invited women were unable to attend or hospitalized when the focus group took place (Table 1). A structured interview guide with four main topics (Table 2) was constructed to answer the primary and secondary aims of this study. Each main topic was introduced and when the discussions between the participants were exhausted, subtopics were used to re-open the discussion by introducing aspects of the topic that had not yet been fully developed.

**Table 1:** Details about the participants in the three focus groups

	Focus group 1 (*n* = 6)	Focus group 2 (*n* = 4)	Focus group 3 (*n* = 4)
Median age years, (range)	65 (63–90)	68 (62–79)	69 (55–85)
Sex (number women)	3 women	2 women	0 women*
Tumor type (rectum/colon)	2 rectum, 4 colon	3 rectum, 1 colon	1 rectum, 3 colon
Median time after surgical treatment, months (range)	27 (9–40)	12 (10–14)	32 (27–40)

* Three women were invited and confirmed attendance but were unable to attend or hospitalized when the focus group took place

**Table 2:** Semi-structured interview guide

Question themes	Aim
Theme 1. Imagine a place	To gain a general understanding of what kind of questions patients would bring to a place (physical or digital), given that they would have had access to a place, to sit down, read, and reflect, since the day of the diagnosis
Theme 2. A digital place	To identify what questions patients prefer to use the Internet to seek answers to, and whether these questions differ from the questions they bring to the health care visit
Theme 3. Regular or occasional visitor	To follow up and gain a more in-depth understanding of different kinds of information needs, depending on whether patients identify themselves as regular or occasional visitors according to the following description: Considering this place we have discussed. Do you want to know as much as possible—the more you now the better it is? (You are a regular visitor). Or do you think it is enough visiting this place if you have a health condition of concern right now. (Occasional visitor)
Theme 4. Coffee meeting	To identify and gain a deeper understanding of what kind of questions patients prefer to discuss with peers, and what questions they think are more relevant to discuss with peers rather than with the health professionals

The focus group interviews were transcribed and analyzed collaboratively through deductive content analysis. First, each researcher independently conducted an analysis, derived from classical content analysis [13]. The second step included a discussion between the authors to compare findings. In the third step, the analysis was discussed and presented for the research team containing both nurses, surgeons, epidemiologists, and information system researchers. The final analytical step was to collaboratively sum up the findings. This included a discussion of each theme and refinements to reach final consensus in the analysis team. Quotes were translated from Swedish to English and edited for ease of reading, but not substantially altered.

## Results

Eighteen patients were invited and fourteen patients participated in the focus groups (those who did not participate were hospitalized or had physical hindrance). Details on the participants are shown in Table 1.

### What type of informational support is requested by the patients?

The patients agreed on the importance of having medical professionals to discuss healthcare issues and treatment options with. The type of informational support that the patients requested differed, depending on individual characteristics and where the patient was in the process of the illness. Regardless, all patients agreed that good communication with healthcare is important. One patient even chose the treatment method which would guarantee the most access and contact with healthcare: “...with the injections as chemotherapy I was here every other week, then I had support … a person that I could talk to who cared for me …” Several patients talked about the initial decisions made in collaboration with healthcare professionals related to the type of surgery, the neoadjuvant and adjuvant treatment. The patients requested information about their treatment and side effects.

The patients also reflected on a hollow space after treatment when communication with healthcare was reduced as treatment was finished. A feeling of being “left alone” by healthcare was described by one of the patients:

What I experienced when I got my diagnosis was that I had acute stomach pain and then I got to know the next day what it was about. But there were a lot of complications and then there was chemotherapy and then I had follow-up for 6 months and then, in a way, I felt quite lonely and abandoned. When the chemo was done, and I had a follow-up, and everything looked great “it looks good, see you later”, but then 6 months went by and I was alone.

When feeling abandoned, there was an expression for a need for contact and communication: “during these 6 months I was recovering both physically and mentally, it would have been great if there had been somewhere, I could turn to.” Furthermore, the patients addressed the need for help in untangling what information is important. As stated by one of the participants, lack of information is not the problem: “You get so much paper that you don’t know what to do with everything.” The problem is getting an overview, as the issue can be information overflow and complexity, and the patients reflected that an online health platform could be of help: “But that’s how it is today, everything is put on the patient. You need to contact this and this and this.” A webpage could be helpful, but it needs to be updated and maintained. The patients asked for information to soothe them in their process, and that a place to find summaries and facts could be calming. One patient emphasized the need for a place to find summaries: “A place where there is information about the disease you have, and then there you can also enter and write patient narratives […] then that would have been a lot better.” Patients reported anxiety from lack of information or information overflow. Not understanding information could even be worse than receiving the diagnosis: “…knowledge calms me down… Worry is the worst thing a patient can have.” One patient also pointed out that reliable information is important for the next-of-kin: “Well, I have a wife who often goes online and read(s).” The patients requested help to discuss changes in bodily functions, the diagnosis itself and advice for discussing with their next-of-kin: “It was really awful for my relatives because I couldn’t answer the questions that they asked me.” The patients clearly indicated a respect for the restricted time the healthcare professionals have but also articulated a need for continued support from healthcare.

### How much information, at what time, given by whom?

How much information the patients felt they needed and how information should be given, changed and evolved during the patient journey. As one patient put it:

When diagnosed, certain information is not important, when all you think about is surviving. But later you wish you would have taken in the information about things that seemed less important then, such as sexuality […] Save your life, with surgery? Cancer or sex? Then nothing is important to me, neither sex nor food. But afterwards when you recover: ‘Yes food is important, yes, sex is important*.*

The granularity of informational support was also discussed, expressed as a need for varying levels of detail in different phases. The patients requested individualized information: “I want this information or I don’t want that information. And maybe, I don’t want it right now, but I would like to have it later … let the patient decide.” The patients also pointed out that the amount of information depended on whether any complications occurred. For example, in an uncomplicated setting less information was needed. One patient summarized this as a fine balance:

“It is probably both, in some situations it may be good to be prepared, but it depends [...] if you get symptoms then you have to do something about it, but if you don’t then you don't want to know about all the other symptoms you could have gotten.”

Some patients asked for detailed information to support decision-making, while another patient opted not to know any details except practical ones before surgery. One patient stated they wanted to know about all possible risks beforehand to prepare: “I have learned that I need to get as much information as possible … I’m searching for information and I want to get so much [information] that I do not miss anything just because one person neglected to tell me.” The patients highlighted the importance of having access to reliable sources of information.

While patients shared experiences of both positive and negative encounters with healthcare personnel, overall, healthcare personnel were described as an important and valuable resource. The nurses were singled out as particularly important: “…they have more time, can answer questions…,” “Nurses are good to talk to ..., you can call and ask them.” The patients referred to the contact nurses as an important stable support link for communication and highlighted the valuable aspects of having a permanent contact. They emphasized that the contact nurse did more than just provide information: “No one told me anything, you had to read about it yourself […] But if you talked to someone […] maybe you can go over it together.” The patients saw the Internet as a possible complementary resource but not a replacement. They stressed the importance of not replacing personal contact with online information. The information received from healthcare personnel was mainly regarding symptoms and treatment alternatives, and was considered reliable. In particular, patients stated the sources on the Internet were difficult to assess reliability, and some were ambivalent towards the Internet due to risks of misinformation. One patient expressed the risk of ending up reading horror stories on various forums: “I’m not the kind of person who uses Google for information ... I ask the questions to those I trust and who have knowledge. I don't want to read a lot of things that can scare me.” Interestingly the patients reported being discouraged to use Google by healthcare professionals.

### What kind of informational support could be given by patients’ peers?

During the focus group sessions, patients identified some topics that were preferably discussed with their peers. This included the practical experience-based tips and tricks from other patients, as well as more psychological-emotional support from a person that also has had cancer. The patients shared information among each other regarding matters of practical nature, based on their experience from everyday life:

Well, just things like if you are going on an airplane - you are entitled to have an extra carry-on bag with you on the flight if you have stoma not just like your regular carry-on bag, you can have your stoma bag with all the accessories so that it is not in the suitcase because the suitcase may disappear.

They addressed that it was difficult to contextualize the information received by healthcare personnel into the meaning of the information regarding practical issues in occurring in everyday life. For instance: “You get a lot of information about the regular chemotherapy side-effects, you know things like hypersensitivity and that the immune system is impaired.” One patient described how this was contextualized when the patient was cleaning at home and hoovering tore away the skin on her hand. She did not understand the practical meaning of sensitivity: “These are things that the doctor could have said to you, ‘be careful now when you are hoovering.”. Another patient exemplified a life-hack of handling stoma leakage when travelling: “I went into a gas station and bought a duct tape. I taped around [shows how she did it], and then it stuck. Yes, that‘s how it is, you occasionally get to be MacGyver.” She told the other participants that she has kept the duct tape roll.

The patients identified that peer support could help them in their psychological process. The patients reflected upon their journey, and saw that they had matured since they received their diagnosis: “It almost felt like I was planning my own funeral [laughs], but now three years later, then I can look back at myself and say ‘oh how stupid I was.’” Even though they have matured and learned, there are critical moments when there is a need for emotional support. The trauma of the cancer can pop up at all hours of the day:

[…] people say time, everything heals with time – no, not so much the things that are buried deep down. And that can pop up when you least expect it. And that's what I mean with support from people who have been there, that has the experience in an uneducated way so to speak, from the non-medical perspective. That can be perceived as different.

Patients described a sense of fellowship when meeting other patients in a similar situation:

“Some websites include patient discussions, and that can be interesting to read about how they [other patients] experience it [...] I have read but I also wrote some posts as well [it’s rewarding] you know you're not alone, but there are many others.”

## Discussion

This study confirms that informational support is important and also shows that information given by healthcare personnel often is good but insufficient. Our study highlights several important needs regarding informational support during and after colorectal cancer treatment. This includes a need to emphasize the importance of nursing and surrounding care, rather than just a reliance on the actual medical treatment [11, 14]. Patients seem to appreciate the psychosocial interventions, but not necessarily by referral to a psychologist for cognitive behavioral therapy.

We have previously shown that patients are generally satisfied with their communication with healthcare personnel, although they have not received information regarding several important side effects of treatment [15]. This study found that patients often feel overwhelmed at the time of diagnosis from the abundance of information received. In addition, patients do not perceive that they have received all information. These results are corroborated by previous studies [9]. As shared decision-making is evolving, it is important to understand what informational support is required in order for the patients to feel sufficiently informed to participate [16]. Furthermore, prior studies have showed that there is potential, not only for shared decision-making but also for shared learning for the healthcare professionals when parts of the care process are digitalized [17–19]. Blended care may be a possible way forward to achieve this type of support.

Informational support include medical matters, the care process and practical issues. This informational support should be pedagogical, and also available to next-of-kin. Previous research highlights that healthcare professionals can play an important role to guide and support patients to navigate online health information [20, 21]. The patients in this study, likewise, indicate that if healthcare can provide reliable sources of information, perhaps they can access information as they seem fit, as long as they have the opportunity to discuss and explore this information with the support of healthcare personnel. The relatives of patients with colorectal cancer also need this kind of informational support. This study illustrates that patients feel that they need improved tools to be able to address their next-of-kin in a more appropriate way. This could likely be achieved by actual meetings with the next-of-kin and encouraging patients to bring their relatives to all hospital and out-patient visits. Still, there is an unmet need for information targeted to relatives. Perhaps the development of communities such as “Patients like me” could be one solution [3]. A healthcare platform with updated information that is reliable and updated continuously could be a way to assist next-of-kin and relieve some burdens from the patient. Patients seem to need information both from healthcare and fellow patients.

Access to informational support is essential, especially as a lack of informational support could create a feeling of being left alone. This was especially evident in the period after treatment. Our findings also show that patients feel abandoned once treatment is finished. Thus, one may conclude that patients desire “reassuring information.” An important observation is that this “soothing information” does not necessarily regard medical issues and may not require an advanced treatment, rather access to peer support and healthcare personnel when needed. As contact nurses are of utmost importance during the treatment phase, perhaps a more individualized and prolonged contact with contact nurses could be a solution. As a part of blended care, contact nurses could also participate in a forum on the Internet. Additionally, our findings suggest that clinical psychology services for patients should be offered on a regular basis as a part of the patient journey.

We also found that the patients believed that a possibility to interact with other patients in the same situation could help them contextualize the knowledge provided by healthcare, share experiences, and practical advice. They even point out that the need for contact with peers is so strong that it could be in some cases more valuable than increased contact with healthcare personnel. The contact requested with peers can entail both the practical and emotional support needed. It appears that healthcare could take advantage of this by creating places in-person or online where patients can meet and discuss all of these matters [22–24]. For example, a health platform that is available 24/7, providing unlimited access to a mix of information and s peer-to-peer support. An online community could support patient empowerment, provide information when it is needed, translating medical knowledge into day-to-day practice, decrease the sense of isolation and increase sense of normalcy, and support emotional well-being [25]. These benefits go hand in hand with the expressed needs found in this study.

The strength of this study includes the recruitment of patients from one center, reducing the effect of center-specific aspects on the results, alongside the qualitative approach which has enabled several new research questions to be posed. The methodology also enables patients to speak from their heart and not only answer constructed questions. Limitations include the limited number of patients. It is of course always difficult to ascertain that saturation in each theme was fully accomplished.

In conclusion, our study shows that the type of informational support that patients with colorectal cancer need depends on individual characteristics and also on the different phases of the care process. This indicates that timing and content are crucial to consider in the process of designing informational support. Our study indicates that we need to focus even more on individualized informational support in the future, and provide options of blended care. Our study also shows that patients, even after finishing treatment, require continued support and express interest in informational support both from healthcare and peers. Patients diagnosed with colorectal cancer need support in discussing aspects related to quality of life (QoL) as they can be affected by complications as well as functional alterations, such as urinary and sexual problems or bowel dysfunction [26–29]. Furthermore, our findings illustrate trends which can be generalized to patients diagnosed with other types of life-altering diseases or chronic conditions, and these aspects relate to discussing emotions and adjusting to behavioral changes in everyday life caused by the condition.

This study has implications for healthcare professionals in several ways: (i) increased understanding of blended care for healthcare professionals has the potential of both increasing quality of care, and increasing self-care, and (ii) increased understanding on how to design informational support can have the dual effect of helping the patients, and moving work from the healthcare professionals. More in-depth studies of online informational support for patients and how digital platforms can facilitate peer support and possibly improve quality of life are needed in order to further complement existing after-care.
